# Albumin overload reduces CFTR and V-ATPase expression and impairs albumin endocytosis and degradation in a renal proximal tubule cell line

**DOI:** 10.3389/fphar.2026.1916632

**Published:** 2026-09-16

**Authors:** Rodrigo Alves da Silva Peres, Carlos Alberto de Souza Filho, Rodrigo P. Silva-Aguiar, Celia Yelimar Palmero Quintana, Raquel Carvalho Castiglione, Maria Oliveira de Souza, Marcelo Marcos Morales, Celso Caruso-Neves, Jackson de Souza-Menezes

**Affiliations:** 1 Grupo de Pesquisa em Fisiologia Aplicada e Comparada, Laboratório Integrado de Ciências Morfofuncionais, Instituto de Biodiversidade e Sustentabilidade NUPEM, Universidade Federal do Rio de Janeiro, Macaé, Brazil; 2 Laboratório: Bioquímica e Sinalização Celular, Instituto de Biofísica Carlos Chagas Filho, Universidade Federal do Rio de Janeiro, Rio de Janeiro, Brazil; 3 Laboratório de Pesquisa Clínicas e Experimentais em Biologia Vascular, Departamento de Ciências Fisiológicas, Instituto de Biologia Roberto Alcantara Gomes, Universidade do Estado do Rio de Janeiro, Rio de Janeiro, Brazil; 4 Laboratório de Fisiologia Renal, Departamento de Fisiologia, Instituto de Ciências Biomédicas, Universidade de São Paulo, São Paulo, Brazil; 5 Laboratório de Fisiologia Celular e Molecular, Instituto de Biofísica Carlos Chagas Filho, Universidade Federal do Rio de Janeiro, Rio de Janeiro, Brazil

**Keywords:** albumin degradation, albuminuria, CFTR, endocytosis, proteinuria, proximal tubule, V-ATPase

## Abstract

**Introduction:**

Albuminuria is a hallmark of kidney disease progression and is associated with impaired glomerular and proximal tubule albumin handling. Efficient receptor-mediated endocytosis and intracellular protein processing in proximal tubule cells require endosomal acidification, a process that depends on multiple transporters, including Cystic Fibrosis Transmembrane Conductance Regulator (CFTR), and its functional interaction with vacuolar H^+^-ATPase (V-ATPase). However, whether albumin overload directly affects CFTR expression and albumin handling in proximal tubule cells remains unclear. Thus, this study aimed to determine the effects of albumin overload on CFTR and V-ATPase expression, as well as on albumin uptake and degradation, using an *in vitro* model of porcine proximal tubule cells (LLC-PK1).

**Methods:**

LLC-PK1 were exposed to low (0.1 mg/mL) or high (20 mg/mL; overload) concentrations of Bovine Serum Albumin (BSA) for 24 or 48 h. CFTR and V-ATPase protein expression were evaluated by Western blotting. Albumin uptake and degradation were assessed using FITC-conjugated BSA (BSA-FITC). CFTR cellular staining was analyzed by confocal immunofluorescence microscopy.

**Results:**

Exposure to 20 mg/mL BSA significantly reduced CFTR expression after both 24 and 48 h, whereas no effects were observed after incubation with a low albumin concentration. V-ATPase expression was significantly reduced only after 48 h of albumin overload. High albumin concentrations also decreased BSA-FITC uptake, with a greater inhibitory effect observed after 48 h of treatment. Furthermore, albumin-overloaded cells exhibited impaired intracellular degradation of endocytosed BSA-FITC, as demonstrated by sustained fluorescence intensity and an increased area under the degradation curve. Confocal microscopy revealed reduced CFTR fluorescence intensity under albumin overload conditions, but no evident alterations in its overall subcellular distribution.

**Conclusion:**

BSA overload coordinately downregulates CFTR and V-ATPase expression and is associated with impaired albumin endocytosis and degradation in proximal tubule cells. These findings reveal a novel association between albumin overload and downregulation of CFTR and V-ATPase proteins in proximal tubule cells. Although the present data are correlative and do not establish a direct causal link between CFTR downregulation and the observed functional defects, they suggest that CFTR/V-ATPase dysregulation may contribute to defective albumin handling under proteinuric conditions and provide a rationale for future mechanistic investigations.

## 1 Introduction

The proximal tubule of the nephron is responsible for the reabsorption of approximately 99% of filtered plasma proteins under physiological conditions, with albumin representing the quantitatively predominant filtered protein at normal glomerular filtration rates (Christensen and Birn, 2001; Devuyst and Ronco, 2023). This highly efficient and selective protein reabsorption system is mediated primarily by receptor-mediated endocytosis through megalin and the complex cubilin/amnionless, transmembrane receptors localized at the apical membrane of proximal tubule epithelial cells (Verroust, 2002; Hall, 2025). Following ligand binding and internalization, albumin and other filtered proteins are transported through early and late endocytic compartments and are ultimately targeted to lysosomes for degradation (Ballabio and Bonifacino, 2020).

Under conditions of glomerular filtration barrier dysfunction, such as those occurring in diabetic nephropathy, IgA nephropathy, focal segmental glomerulosclerosis, and other proteinuric diseases, the increased passage of albumin through the glomerular capillary barrier substantially increases the albumin load reaching the proximal tubule lumen. This tubular albumin overload has been recognized as a key mediator of tubulointerstitial injury, inflammation, and fibrosis, contributing to the progressive loss of renal function (Zoja et al., 1995; 2003; Abbate et al., 2002; 2006; Donadelli et al., 2003; Perico et al., 2008; Cortinovis et al., 2024; Makhammajanov et al., 2024). Indeed, pathophysiological concentrations of albumin have been shown to alter the luminal pH of endosomal compartments, leading to reduced albumin uptake by proximal tubule cells (Gekle et al., 1995; Ishibashi, 2006). However, the cellular mechanisms underlying these alterations in endosomal pH remain poorly understood.

The Cystic Fibrosis Transmembrane Conductance Regulator (CFTR), best known as a chloride channel, has emerged as a pleiotropic regulator of epithelial cell function beyond its role in ion transport (Cutting, 2015). CFTR is localized to the apical membrane and endocytic compartments of polarized epithelial cells, including those of the renal proximal tubule (Pranke and Sermet-Gaudelus, 2014), where it contributes to the regulation of endosomal pH through functional coupling with vacuolar H^+^-ATPase (V-ATPase) (Jouret et al., 2011; Liu et al., 2012; Devuyst and Luciani, 2015). In addition, CFTR has been reported to regulate both the expression and activity of V-ATPase in proximal tubule cells. (Carraro-Lacroix et al., 2010).

The present study aimed to investigate the relationship between high albumin concentrations and the expression of CFTR and V-ATPase, as well as albumin uptake and degradation capacity, in an *in vitro* model of porcine proximal tubule epithelium (LLC-PK1 cells). We hypothesized that albumin overload would reduce CFTR and V-ATPase expression and impair the capacity of proximal tubule cells to internalize and degrade albumin. Our findings provide new insights into the potential contribution of CFTR dysregulation to impaired proximal tubule albumin handling.

## 2 Materials and methods

### 2.1 Cell culture and experimental design

LLC-PK1 cells (*Sus scrofa* proximal tubule cell line, ATCC CL-101^TM^) were cultured in Dulbecco’s Modified Eagle Medium (DMEM with 1,000 mg/L glucose and L-glutamine; Sigma-Aldrich D5523) supplemented with 3.7 g/L sodium bicarbonate, 5% fetal bovine serum (FBS; Cultilab 000063), and antibiotic-antimycotic solution 1X containing penicillin (100 IU/mL), streptomycin (100 μg/mL), and amphotericin B (0.25 μg/mL) (Sigma-Aldrich A5955). Cells were maintained at 37 °C in a humidified atmosphere of 5% CO_2_ (Sanyo MCO-17AC incubator) and passaged every 3 days. All culture media and reagents were verified to be endotoxin-free prior to use.

For experimental conditions, confluent LLC-PK1 cells were exposed to three treatment groups, each applied for either 24 or 48 h:

1. CTRL: DMEM (standard culture condition)
2. ALB 0.1: DMEM with 0.1 mg/mL of bovine serum albumin (BSA; Millipore 81-053-3)
3. ALB 20: DMEM with 20 mg/mL of BSA

The 0.1 mg/mL concentration was selected as a basal, non-overloading reference condition, consistent with its use in similar *in vitro* proximal tubule studies (Gekle et al., 1995; Ishibashi, 2006), though it should be noted that precise *in vivo* measurements of albumin concentration in the proximal tubular lumen are technically challenging and not directly available.

The 20 mg/mL albumin concentration was selected to simulate proteinuric stress encountered in advanced nephropathy, wherein proximal tubule cells are exposed to markedly elevated filtered protein loads (Abbate et al., 2006). This *in vitro* albumin overload model is intended to simulate the tubular consequences of elevated albumin filtration that occurs in the context of glomerular barrier dysfunction, as described in experimental and clinical studies of proteinuric kidney disease (Zoja et al., 1995; 2003; Abbate et al., 2002; Donadelli et al., 2003; Perico et al., 2008).

Cells were seeded in 6-well plates at a density of 5 × 10^4^ cells/well and cultured for 24 h under the previously described culture conditions until reaching approximately 70% confluence. Subsequently, the cells were subjected to the experimental conditions. For all experimental groups, cells were maintained in DMEM supplemented with 10% FBS and were serum-starved during the final 6 h preceding the end of each treatment period (24 and 48 h).

To determine whether albumin overload affects cell viability, two cell viability assays were performed using the Trypan Blue exclusion method. In the first assay, cell viability was assessed during cell growth while cells were continuously exposed to 20 mg/mL albumin for up to 72 h. In the second assay, cell viability was evaluated following a 48 h treatment with 20 mg/mL albumin, as described previously.

### 2.2 BSA-FITC endocytosis and degradation assay

For this assay, bovine serum albumin (BSA) conjugated to fluorescein isothiocyanate (FITC) (BSA-FITC, A9771, Sigma-Aldrich) was used. FITC is a pH-sensitive fluorophore widely employed in cell and molecular biology assays to visualize the trafficking and transport of conjugated molecules, including albumin (Gekle et al., 1995; Ohgaki et al., 2017). Based on the pH-dependent fluorescence properties of FITC and the trafficking of albumin through endolysosomal compartments exhibiting progressive acidification following endocytosis, it was assumed that the decay in FITC fluorescence could serve as a relative measure of BSA-FITC degradation within acidic intracellular compartments.

LLC-PK1 cells were cultured in 24-well plates under the same experimental conditions previously described. At the end of the incubation period for each experimental condition, cells were washed with 1× phosphate-buffered saline (PBS; pH 7.4; 136 mM NaCl, 2.7 mM KCl, 8 mM Na2HPO4.7H2O, and 1.76 mM KH2PO4) and incubated for 30 min at 37 °C in DMEM containing 50 μg/mL BSA-FITC. This incubation period represented the uptake phase of BSA-FITC by LLC-PK1 cells.

Following the 30-min uptake period, cells were washed five times with 1× Ringer solution (pH 7.4; 140 mM NaCl, 2.7 mM KCl, 1.8 mM CaCl2, 1 mM MgCl2, 5.5 mM D-glucose, and 12.4 mM HEPES-Tris) to remove excess extracellular BSA-FITC. After removal of unbound BSA-FITC, cells were maintained in 1× Ringer solution at 37 °C to allow intracellular processing of the endocytosed molecules for the designated time intervals of 0, 5, 15, and 30 min. At each time point, cells were washed twice with 1× Ringer solution (pH 7.4) and lysed using 1× lysis buffer (20 mM MOPS, 0.1% Triton X-100, pH 7.4). The 0-min time point corresponds to the amount of BSA-FITC internalized during the uptake phase and therefore represents the initial level of albumin uptake prior to intracellular processing and degradation.

Cell lysates were homogenized and transferred to 96-well plates for fluorescence measurements. Fluorescence was quantified using a PerkinElmer 2030 Multilabel Reader VICTOR X2 fluorimeter with an excitation wavelength of 495 nm. Cellular autofluorescence was determined and subtracted from the total fluorescence measured in each experimental condition. Total protein concentration was determined using the Lowry method (Lowry et al., 1951) and used to normalize fluorescence intensity in each sample.

### 2.3 Protein extraction and western blot analysis

At the end of each experiment, total cellular proteins were extracted using 150 μL of 1× lysis buffer containing 20 mM HEPES, 2 mM EGTA (pH 7.4), 50 mM sodium fluoride, 5 mM sodium pyrophosphate, 5 mM sodium orthovanadate, and 10 mM β-glycerophosphate, supplemented with a protease inhibitor cocktail (Sigma-Aldrich). Cell lysates were collected, gently agitated for 30 min, and centrifuged at 10,000 × *g* for 10 min at 4 °C. The resulting supernatants, containing total cellular proteins, were collected and used for Western blot analysis.

Total protein concentration was determined according to the Lowry method as described above.

All protein samples were diluted to a final concentration of 2 μg/μL in sample buffer containing 50% glycerol, 1 M Tris-HCl (pH 6.8), 10% sodium dodecyl sulfate (SDS), 4 mg bromophenol blue, and 5% β-mercaptoethanol, and then heated at 100 °C for 5 min.

A total of 50 μg of protein from each sample was subjected to denaturing electrophoresis on 9% polyacrylamide gels. Electrophoresis was performed at a constant voltage of 170 V for 60 min in running buffer containing 25 mM Tris, 192 mM glycine, and 0.1% SDS.

Following electrophoresis, proteins were transferred onto polyvinylidene fluoride (PVDF) membranes (Millipore) using transfer buffer containing 25 mM Tris, 192 mM glycine, and 15% methanol. Protein transfer was carried out at a constant current of 380 mA for 60 min. Membranes were then blocked for 3 h in Tris-buffered saline containing 0.05% Tween-20 (TBS-T, pH 7.4) supplemented with 5% nonfat dry milk. After blocking, membranes were washed with 1× TBS-T and incubated with primary antibodies.

The primary antibodies used were anti-CFTR (CF3, ab2784, Abcam), anti-V-ATPase subunit E (ab50208, Abcam), and anti-β-actin (ab8227, Abcam). Horseradish peroxidase-conjugated anti-rabbit IgG (Cell Signaling Technology #7074) and anti-mouse IgG (Cell Signaling Technology #7076) were used as secondary antibodies. Membranes were incubated with primary antibodies for approximately 16 h at 4 °C in 1× TBS-T containing 5% BSA. Primary antibodies were diluted at 1:1,000 for anti-CFTR, anti-V-ATPase, and anti-β-actin. Membranes were subsequently washed with 1× TBS-T before incubation with secondary antibodies.

Secondary antibody incubation was performed for 1 h in 1× TBS-T containing 5% nonfat dry milk at a dilution of 1:5,000. After incubation, membranes were washed with 1× TBS-T for approximately 40 min, with the washing solution replaced every 5 min. Membranes were then maintained in 1× TBS until signal detection.

Protein bands were visualized by chemiluminescence using the Amersham ECL Prime Detection Kit (Life Sciences), which contains a peroxidase substrate. Images were acquired using the ImageQuant LAS 4000 imaging system (GE Healthcare Life Sciences). Band densitometry (pixels/mm^2^) was quantified using Fiji ImageJ software. Relative protein expression was determined by normalizing the densitometric value of each target protein band to the densitometric value of the corresponding β-actin band, which served as the internal loading control. Results were expressed as fold changes in protein expression relative to the control group.

### 2.4 Immunofluorescence assay

For CFTR immunofluorescence analysis, the monoclonal anti-CFTR antibody CF3 (ab2784, Abcam) was used. LLC-PK1 cells were cultured on circular glass coverslips placed in 24-well plates for 48 h under the experimental conditions previously described. Following the incubation period, cells were maintained at 4 °C and washed with 1× phosphate-buffered saline (PBS; pH 7.4; 136 mM NaCl, 2.7 mM KCl, 8 mM Na2HPO4.7H2O, and 1.76 mM KH2PO4).

Cells were then fixed with 4% paraformaldehyde (PFA) for 15 min. Membrane permeabilization was performed by incubating the cells in 0.1% Triton X-100 (Sigma-Aldrich) for 15 min. Following five washes with 1× PBS, cells were incubated for 1 h at room temperature under gentle agitation in blocking solution containing 5% BSA in 1× PBS.

Cells were subsequently incubated with the monoclonal anti-CFTR CF3 primary antibody according to the manufacturer’s recommendations at a dilution of 1:100 in 5% BSA prepared in 1× PBS. Primary antibody incubation was performed for 1 h at room temperature. Cells were then washed five times with 1× PBS to remove excess primary antibody and subsequently incubated with the secondary antibody.

The secondary antibody solution consisted of Alexa Fluor 488-conjugated goat anti-mouse IgG (A21202, Thermo Fisher Scientific) diluted 1:200 in 5% BSA prepared in 1× PBS. After 1 h of incubation with the secondary antibody, cells were washed five times with 1× PBS and stained with DAPI (D1306, Thermo Fisher Scientific) at a final concentration of 1 μg/mL for 15 min to visualize cell nuclei. Following DAPI staining, cells were washed five times with 1× PBS.

Coverslips were mounted onto glass microscope slides using VECTASHIELD mounting medium (H-1000, Vector Laboratories). Images were acquired using a Leica TCS SP8 confocal microscope (Leica Microsystems) operated with LAS X Life Science software. Image processing and analysis were performed using Fiji ImageJ software.

### 2.5 Statistical analysis

All experiments were performed in duplicate with a minimum of three independent replicates (separate cell passages). Data are presented as mean ± standard error of the mean (SEM). Normality of data distribution was assessed using the Shapiro-Wilk test. For comparisons among multiple groups, two-way analysis of variance (Two-Way ANOVA) was performed with Sidak’s multiple comparisons test. For pairwise comparisons between two groups, unpaired Student’s t-test was used. Differences were considered statistically significant at P < 0.05. All statistical analyses were performed using GraphPad Prism version 9.0 (GraphPad Software, San Diego, CA).

### 2.6 Ethical considerations

This study utilized established mammalian cell lines (LLC-PK1) obtained from the American Type Culture Collection and did not involve primary tissues or animals. No institutional animal care and use committee (IACUC) approval was required. All reagents were used in accordance with manufacturers’ specifications and relevant laboratory safety guidelines.

## 3 Results

### 3.1 Albumin overload coordinately downregulates CFTR and V-ATPase expression without affecting cell viability

Cell viability was assessed under albumin overload during cell growth for up to 72 h and after a 48 h treatment, as described previously. No significant differences in cell viability were observed in either assay (Supplementary Figure S1).

Albumin overload in the tubular lumen is known to induce a variety of alterations in proximal tubule cell physiology (Gekle, 2005; Abbate et al., 2006; Birn and Christensen, 2006). To investigate the potential effects of albumin overload on the regulation of CFTR expression in proximal tubule cells, LLC-PK1 cells were exposed to 20 mg/mL BSA Considering the presence of albumin at low concentrations within the proximal tubular fluid, LLC-PK1 cells were also cultured in medium supplemented with 0.1 mg/mL BSA for 24 and 48 h.

Cells exposed to a high concentration of albumin (20 mg/mL BSA) for 24 h resulted in significantly lower CFTR expression (Figures 1A,C; Table 1), which persisted at 48 h (Figures 1B,D; Table 1). CFTR expression did not differ significantly between the BSA 0.1 and CTRL groups at both time point (Figures 1A–D; Table 1).

**FIGURE 1:**
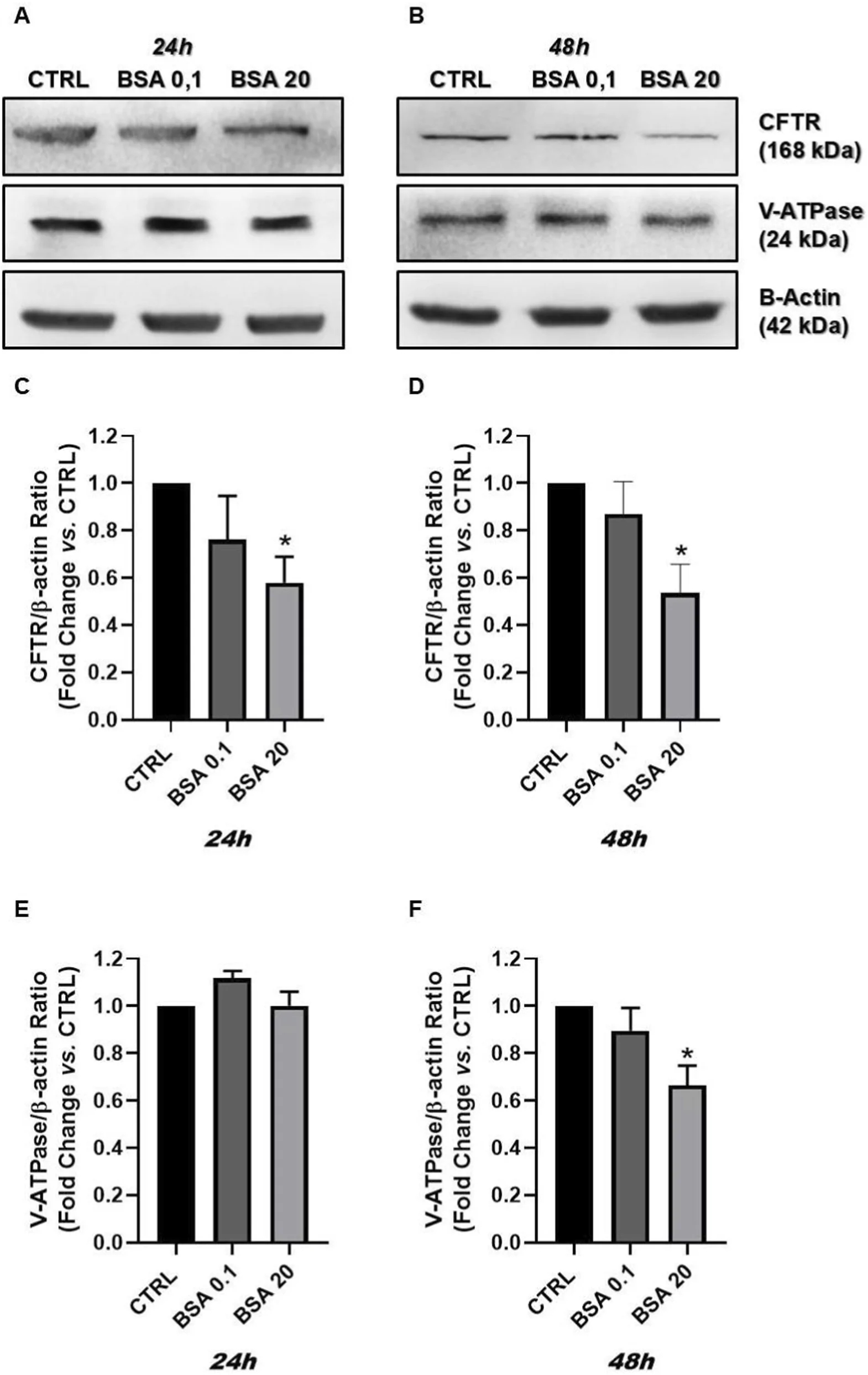
Relative expression of CFTR and V-ATPase in LLC-PK1 cells [exposed to low-dose] (BSA 0.1 mg/mL) and high (BSA 20 mg/mL) albumin concentrations for 24 h and 48 h. **(A,B)** Representative Western blot images showing the detection of CFTR, V-ATPase, and β-actin on PVDF membranes. **(C,D)** Bar graphs showing the relative densitometric quantification of CFTR expression after 24 h and 48 h of treatment, respectively. **(E,F)** Bar graphs showing the relative densitometric quantification of V-ATPase expression after 24 h and 48 h of treatment, respectively. Data are presented as mean ± standard error of the mean (SEM) from three independent experiments (n = 6). Statistical significance was considered at p < 0.05 (*). β-Actin was used as the loading control.

**TABLE 1:** Summary of the results obtained from descriptive statistical analyses and hypothesis testing.

Parameter	24 hours			48 hours			​
	CTRL mean ± SEM 95% CI	BSA 0.1 mean ± SEM *95% CI* *p-value*	BSA 20 mean ± SEM *95% CI* *p-value*	CTRL mean ± SEM *95% CI*	BSA 0.1 mean ± SEM *95% CI* *p-value*	BSA 20 mean ± SEM *95% CI* *p-value*	Sample Size (n)
CFTR/β-Actin (Fold Change)	1	0.76 ± 0.18 0.25 – 1.27 0.35	0.58 ± 0.11* 0.29 – 0.86 0.04	1	0.87 ± 0.14 0.52 – 1.22 0.66	0.54 ± 0.12* 0.23 – 0.85 0.02	6
V-ATPase/β-Actin (Fold Change)	1	1.12 ± 0.03 1.02 – 1.21 0.16	1.00 ± 0.06 0.85 – 1.15 0.99	1	0.89 ± 0.10 0.66 – 1.13 0.58	0.66 ± 0.08* 0.47 – 0.86 0.01	6
CFTR (CTCF Fold Change)	–	–	–	1	–	0.53 ± 0.08* 0.29 – 0.78 0.01	4
Albumin UpTake (Fold Change)	1 ± 0.00 0.99 – 1.01	–	0.86 ± 0.01* 0.83 – 0.88 < 0.0001	1 ± 0.04 0.91 – 1.09	–	0.56 ± 0.04* 0.43 – 0.69 < 0.0001	6
Albumin Degradation (AUC)	–	–	–	9.57 ± 1.28 7.05 – 12.08	–	14.76 ± 1.08* 12.63 – 16.88 0.01	6

*p < 0.05.

The expression and activity of V-ATPase in the endosomal membrane are essential for the acidification of the endosomal lumen and, consequently, for the processing and intracellular trafficking of endocytosed molecules (Marshansky et al., 2002; Forgac, 2007; Scott and Gruenberg, 2011). Considering that both the activity and expression of V-ATPase have been shown to depend on CFTR expression in proximal tubule cells (Carraro-Lacroix et al., 2010), the expression of this protein was evaluated under conditions in which reduced CFTR expression was observed. In this context, V-ATPase expression was analyzed in LLC-PK1 cells cultured in the presence of BSA for 24 and 48 h.

After 48 h of treatment, cells in the BSA 20 group exhibited significantly lower V-ATPase expression than those in the CTRL and BSA 0.1 groups (see Figures 1B,F; Table 1). In contrast, there was no significant difference in V-ATPase expression among the experimental groups after 24 h of treatment. (Figures 1A,E; Table 1).

### 3.2 Albumin overload impairs BSA-FITC endocytosis

The effect of albumin overload on albumin endocytosis was evaluated by measuring BSA-FITC uptake after 24 and 48 h of treatment.

BSA-FITC uptake was significant lower in cells exposed to 20 mg/mL BSA at both time points (Figures 2A,B; Table 1). Moreover, the lower endocytic capacity was more pronounced after 48 h than after 24 h of treatment, suggesting a progressive impairment of albumin endocytosis in response to sustained albumin overload (Figure 2C).

**FIGURE 2:**
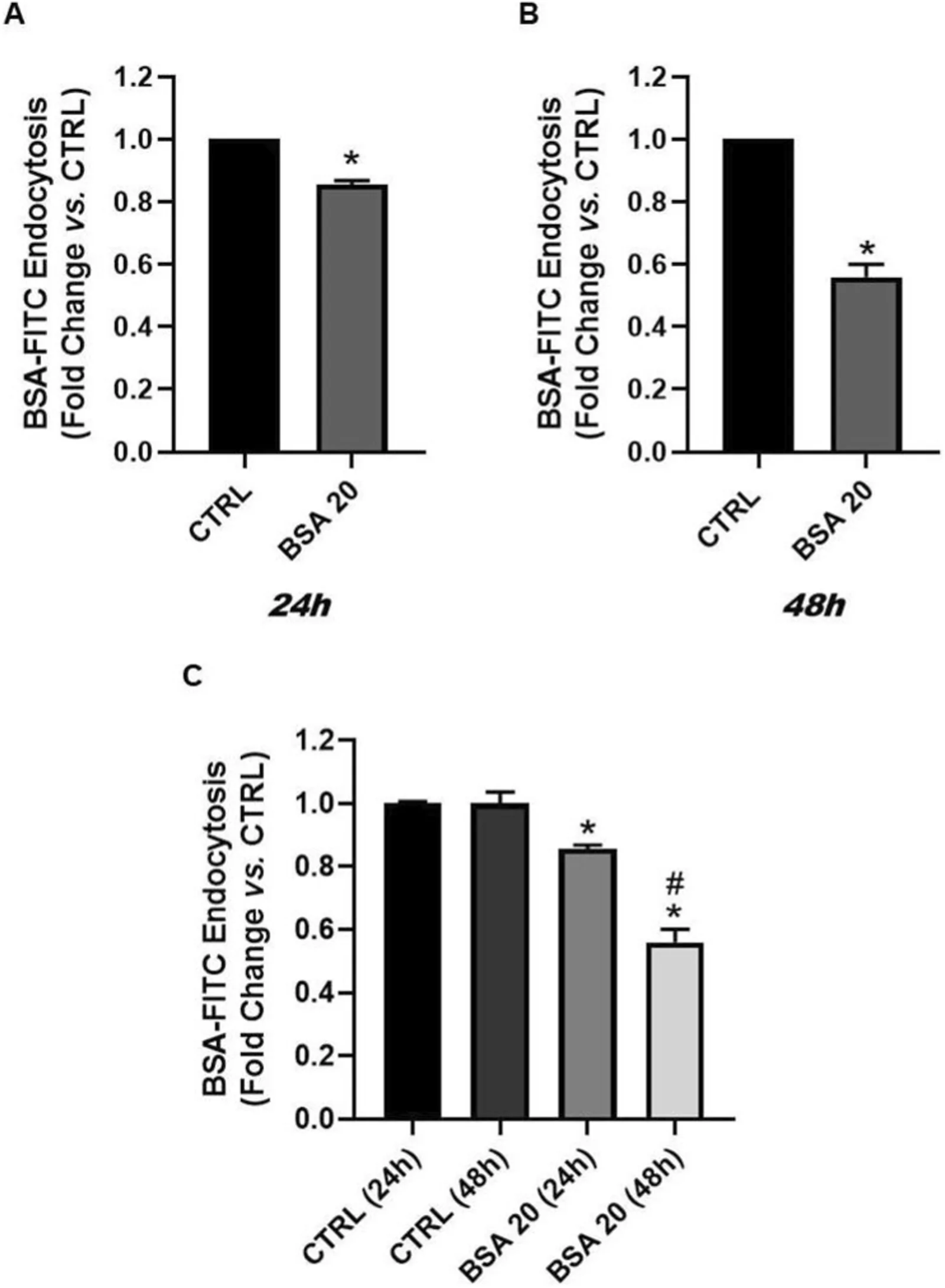
BSA–FITC uptake in LLC-PK1 cells exposed to a high albumin concentration (BSA 20 mg/mL) for 24 h and 48 h. **(A,B)** Bar graphs showing relative BSA–FITC uptake after 24 h and 48 h of treatment, respectively. **(C)** Two-way analysis of variance demonstrating a significant difference between the BSA 20 (24 h) and BSA 20 (48 h) groups. Data are presented as mean ± standard error of the mean (SEM) from three independent experiments (n = 6). Statistical significance was considered at p < 0.05 (*, #). (*) Significant difference compared with the CTRL (24 h) and CTRL (48 h) groups. (#) Significant difference between the BSA 20 (24 h) and BSA 20 (48 h) groups.

### 3.3 Albumin overload impairs intracellular albumin degradation

Following endocytosis by proximal tubule cells, albumin molecules are predominantly targeted to lysosomes for intracellular degradation, followed by the release of their catabolic products into the extracellular medium (Ohshita and Hiroi, 1998; Gudehithlu et al., 2004; Gekle, 2005). While the effects of pathophysiological albumin concentrations on receptor-mediated endocytosis have been investigated in proximal tubule cells, the impact of albumin overload on the degradation of endocytosed albumin has received considerably less attention (Caruso-Neves et al., 2006; De Barros Peruchetti et al., 2018).

Therefore, we investigated whether proximal tubule cells exposed to 20 mg/mL albumin for 48 h (a condition associated with lower CFTR and V-ATPase expression and more pronounced BSA-FITC uptake) exhibited impaired albumin degradation capacity. Analysis of BSA-FITC fluorescence decay revealed a slower reduction in fluorescence intensity throughout the degradation time course in cells exposed to high albumin concentrations compared with control cells (Figure 3A). Specifically, fluorescence intensity remained significantly higher in the BSA 20 group at all evaluated time points (5, 15, and 30 min), indicating reduced intracellular degradation of BSA-FITC.

**FIGURE 3:**
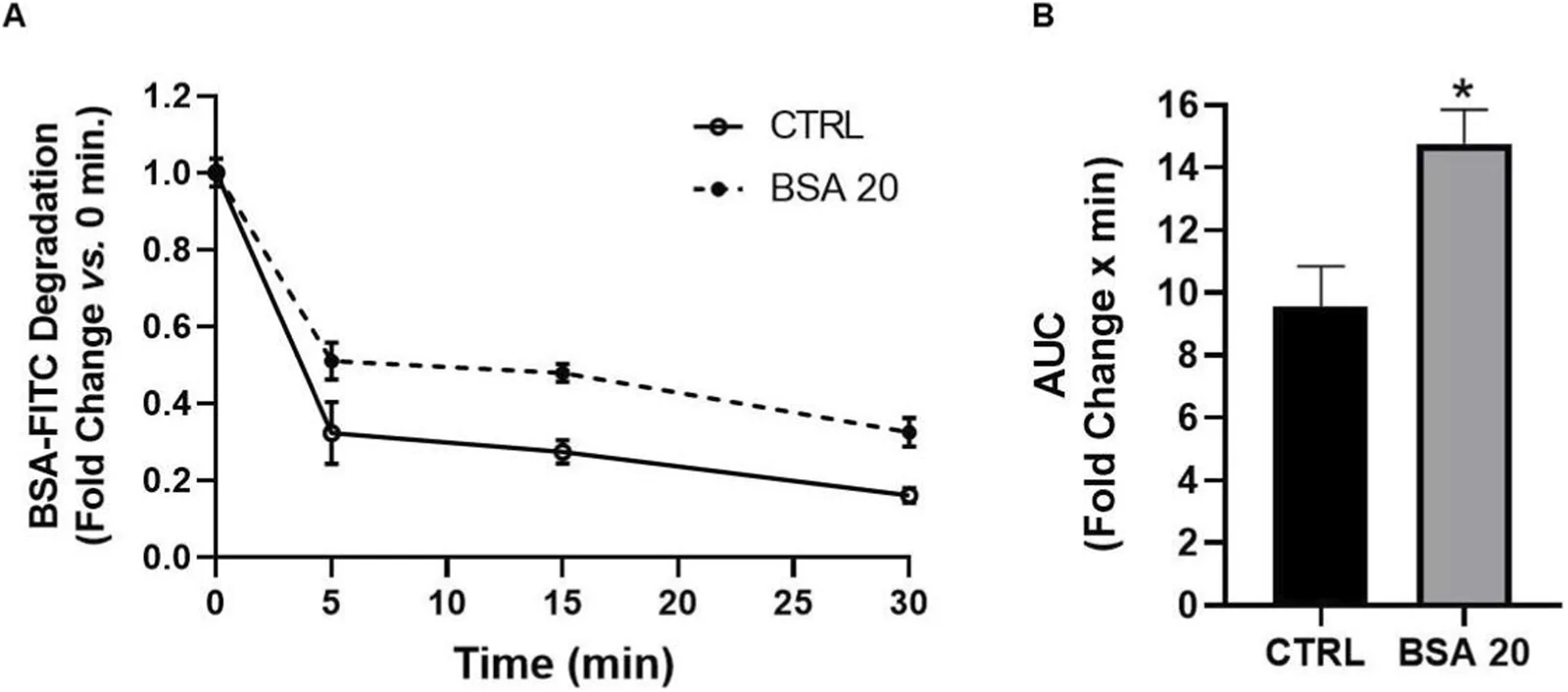
Time course of BSA-FITC degradation and area under the curve AUC in LLC-PK1 cells exposed to a high albumin concentration (BSA 20 mg/mL) for 48 h. **(A)** Cells were incubated with BSA–FITC for 30 min at 37 °C to allow endocytosis. Time zero represents the time point immediately after the uptake period and corresponds to the total amount of BSA–FITC endocytosed under each experimental condition. Subsequent time points represent the degradation of the initially endocytosed BSA–FITC. Data are representative of three independent experiments (n = 6). **(B)** Area under the curve (AUC) representing the relative fluorescence intensity normalized to the initial endocytic uptake throughout the time course of BSA–FITC degradation. The bar graph depicts the total AUC for each experimental condition. Data are presented as mean ± standard error of the mean (SEM). Statistical significance was considered at p < 0.05 (*).

Consistent with these findings, analysis of the area under the curve (AUC) demonstrated a greater cumulative fluorescence signal throughout the degradation period in the BSA 20 group compared with the CTRL group (Figure 3B; Table 1). These results indicate that albumin overload impairs the intracellular degradation of endocytosed albumin in proximal tubule cells.

### 3.4 Subcellular localization of CFTR under high albumin conditions

Considering that CFTR is an integral membrane protein with an important intracellular distribution and that its subcellular localization is associated with distinct cellular functions (Bradbury, 1999; Jouret et al., 2011), we investigated whether exposure to a high albumin concentration (20 mg/mL), for 48 h (a condition associated with lower CFTR and V-ATPase expression and more pronounced BSA-FITC uptake), would promote not only changes in CFTR protein expression but also alterations in its subcellular localization in LLC-PK1 cells. To address this question, CFTR localization was evaluated by laser scanning confocal immunofluorescence microscopy using a specific anti-CFTR antibody.

Initially, CFTR was found to exhibit a diffuse cellular distribution, being detected throughout the cytoplasm under all experimental conditions (Figure 4A). Furthermore, analysis of the XZ optical sections revealed that CFTR was localized both at the plasma membrane and within the cytoplasm (Figure 4B). Consistent with the Western blot results, a lower CFTR fluorescence intensity was observed in cells exposed to the high albumin concentration, indicating reduced CFTR expression under this condition (Figure 4C).

**FIGURE 4:**
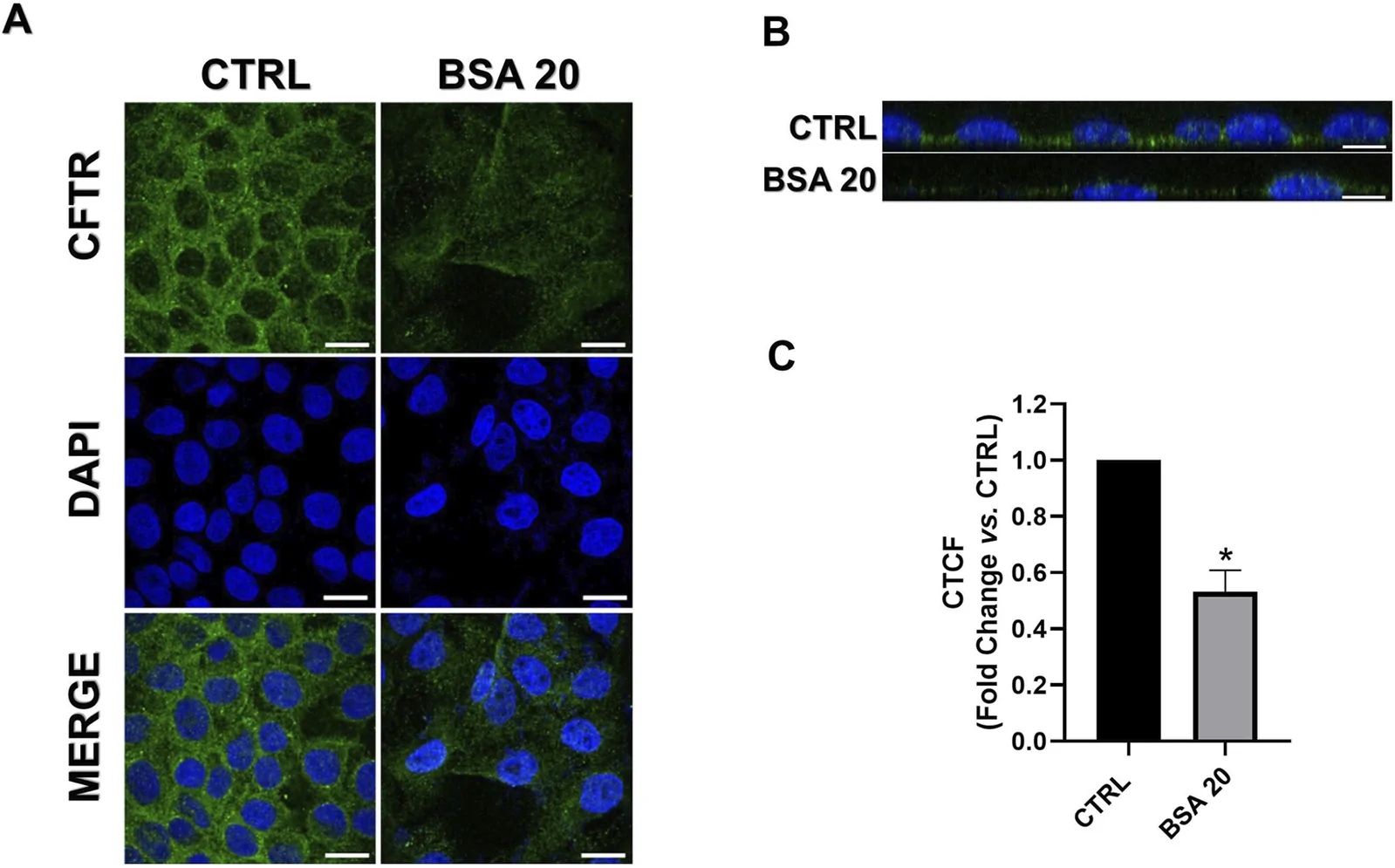
Immunofluorescence analysis of CFTR in LLC-PK1 cells exposed to a high albumin concentration (BSA 20 mg/mL) for 48 h. **(A)** CFTR immunostaining is shown in green, whereas nuclei stained with DAPI are shown in blue. Merged images showing the overlap between CFTR and DAPI staining are presented for each experimental condition. Images were acquired using a confocal laser scanning microscope at ×60 magnification. Scale bar = 20 μm. **(B)** XZ optical sections showing the merged CFTR (green) and DAPI (blue) fluorescence signals for each experimental condition. Scale bar = 10 μm. **(C)** Corrected Total Cell Fluorescence (CTCF) Data are presented as mean ± SEM from [n] cells from [n] independent experiments. Statistical significance was considered at p < 0.05.

## 4 Discussion

The present study was designed as an initial observational investigation to explore whether albumin overload is associated with alterations in CFTR expression in LLC-PK1 cell line. Accordingly, the findings should be interpreted as preliminary and hypothesis-generating rather than as evidence of a causal relationship.

LLC-PK1 cells were selected for the present study because this cell line expresses megalin-mediated endocytosis, CFTR, and a functional endolysosomal pathway (Nielsen et al., 1998; Carraro-Lacroix et al., 2010), and has been extensively validated for studies of albumin endocytosis and BSA-FITC-based assays of lysosomal function in the proximal tubule context (Gekle et al., 1995; Ishibashi, 2006). Importantly, porcine proximal tubular cells share strong structural and functional homology with human proximal tubule cells. Nevertheless, given the increasing availability of human proximal tubule cell models (e.g., HK-2, ciPTEC), future studies should validate the current findings in human-derived cellular systems to assess the translational relevance of the present observations.

Importantly, the BSA preparation used in this study (Millipore 81-053-3) contains a low fatty acid content. Published evidence indicates that, in contrast to fatty acid-loaded albumin, exposure of proximal tubule cells to high concentrations of fatty acid-free BSA induces minimal overt cytotoxicity while still disrupting endosomal processing (Ruggiero et al., 2014; Long et al., 2020). Consistent with these observations, no significant changes in cell viability were detected in either of the Trypan Blue exclusion assays performed in the present study (Supplementary Figure S1). Although Trypan Blue exclusion assay reliably detects the loss of plasma membrane integrity, it does not distinguish between different modes of cell death or identify early stages of apoptosis. Therefore, future studies should incorporate complementary assays, such as lactate dehydrogenase (LDH) release and annexin V/propidium iodide staining, to provide a more comprehensive evaluation of cell viability and to exclude potential cytotoxic effects of albumin overload.

The effects of albumin overload on proximal tubule cell physiology have been investigated as a model of the tubular response to increased glomerular protein filtration. In the clinical setting, tubular albumin overload is not a primary event but rather the downstream consequence of glomerular filtration barrier dysfunction, as occurs in diabetic nephropathy, IgA nephropathy, focal segmental glomerulosclerosis, and other proteinuric diseases (Zoja et al., 1995; 2003; Abbate et al., 2002; 2006; Morigi et al., 2002; Donadelli et al., 2003). The *in vitro* model used in the present study should therefore be interpreted as representative of events occurring in the tubular compartment as a consequence of pre-existing or concomitant glomerular disease. Here, it was employed a two-dose experimental design (0.1 mg/mL as a low/basal condition and 20 mg/mL as a pathophysiological overload condition), consistent with established paradigms in the literature (Abbate et al., 2006; Lee et al., 2013; Chen et al., 2017). An important limitation is that a systematic dose-response analysis covering intermediate albumin concentrations (e.g., 2, 5, and 10 mg/mL) was not performed. Such experiments would be valuable to determine the minimum effective concentration for CFTR and V-ATPase downregulation, to assess whether these effects develop progressively or exhibit a threshold behavior, and to establish dose-dependent relationships between albumin exposure and endocytic function. Future studies should include a comprehensive dose-response analysis, together with formal assessment of cell viability at each concentration point.

To our knowledge, this is the first study to demonstrate that exposure to albumin overload reduces CFTR expression in LLC-PK1 proximal tubule cells. Our findings suggest that the mechanism underlying the reduction in total CFTR expression may be associated with albumin overload of the receptor-mediated endocytic machinery, since low albumin concentration did not affect CFTR expression. A limitation of the present study is that the expression of megalin, cubilin, and ClC-5, the primary receptor and chloride/proton transporter involved in apical albumin endocytosis in the proximal tubule, was not assessed under albumin overload conditions. These proteins are essential components of the receptor-mediated endocytic machinery and have been shown to be downregulated in proteinuric states (Figueira et al., 2017; Fatah et al., 2018; Trimarchi et al., 2021). Their potential downregulation could independently contribute to, or interact with, the CFTR/V-ATPase axis alterations described here in determining the observed endocytic impairment. Future studies should include parallel assessment of megalin, cubilin, and ClC-5 expression and trafficking under albumin overload conditions to provide a more comprehensive characterization of the endocytic defect. Also, the assessment of CFTR or V-ATPase mRNA expression was not performed. Therefore, whether albumin overload regulates CFTR expression at the transcriptional or post-transcriptional/post-translational level remains to be established. CFTR protein expression can be reduced by increased endoplasmic reticulum-associated degradation or proteasomal turnover without changes in mRNA levels (Pranke and Sermet-Gaudelus, 2014). Future experiments incorporating RT-qPCR analysis of CFTR and V-ATPase mRNA, along with proteasome inhibitor studies, will be important to define the molecular mechanism underlying albumin overload-induced CFTR downregulation.

Endosomal acidification is an essential step in the intracellular processing of endocytosed cargo in proximal tubule cells (Nielsen et al., 1998; Marshansky et al., 2002; Forgac, 2007; Christensen et al., 2009; Elkin et al., 2016). This process depends largely on the activity of V-ATPase (Christensen et al., 2003; Tenten et al., 2013; Dickson et al., 2014; Elkin et al., 2016). In addition, chloride influx into the endosomal lumen mediated by both ClC-5 and CFTR contributes to endosomal acidification (Jentsch, 2007; Devuyst and Luciani, 2015; Elkin et al., 2016). Alterations in endosomal pH induced by V-ATPase inhibition have been shown to reduce albumin-FITC uptake in proximal tubule cells (Gekle et al., 1995; Gekle, 2005).

Previous studies using cystic fibrosis animal models demonstrated that these animals exhibit defects in apical receptor-mediated endocytosis associated with reduced proximal tubular expression of cubilin (Jouret et al., 2007). The same study also reported increased urinary excretion of β2-microglobulin and transferrin in patients with cystic fibrosis, both of which are proteins predominantly reabsorbed by the proximal tubule (Jouret et al., 2007). Similar findings were obtained by our research group, which demonstrated that rats with type 1 diabetes mellitus exhibited reduced cortical cubilin expression, accompanied by decreased CFTR expression and increased urinary transferrin excretion (Figueira et al., 2017). Another study of our group demonstrated that CFTR silence in rat proximal tubule cell line results in reduced V-ATPase activity and expression (Carraro-Lacroix et al., 2010). Based on the reduction in CFTR expression observed under high-albumin conditions in this study, we hypothesized that V-ATPase expression would also be decreased. Our results showed that exposure to 20 mg/mL BSA for 48 h significantly reduced the expression of the E subunit of V-ATPase. Furthermore, the coordinated downregulation of CFTR and V-ATPase, induced by albumin overload, appeared to be time-dependent, since reductions in both expressions were observed only after 48 h.

In addition to its expression level, the cellular localization of CFTR is critical for its physiological functions (Bradbury et al., 1992; Kleizen et al., 2000; Ameen et al., 2007). CFTR is known to localize both to the plasma membrane and to intracellular compartments, where it performs distinct functions depending on its subcellular localization (Bradbury et al., 1992; Bradbury, 1999). Immunofluorescence analysis revealed that CFTR maintained a diffuse distribution pattern, being detected both at the plasma membrane and within intracellular compartments. These findings are consistent with the cellular distribution pattern previously reported in other proximal tubule cell lines (Carraro-Lacroix et al., 2010). Thus, the cellular alterations mediated by CFTR in response to albumin overload are unlikely to result from changes in its overall subcellular localization. Nevertheless, future co-localization studies using compartment-specific markers such as Rab5 (early endosomes), Rab7 (late endosomes), and LAMP1 (lysosomes) would provide direct evidence of whether albumin trafficking through the endolysosomal pathway is altered following albumin overload (Shipman et al., 2023).

Lee and colleagues (2013) investigated the degradation of receptor-mediated endocytic cargo using Alexa-conjugated albumin (BSA-Alexa) and retinol-binding protein (RBP-Alexa) in proximal tubules from mice subjected to albumin overload (Lee et al., 2013). Consistent with our findings, the authors demonstrated by confocal microscopy that albumin overload resulted in intracellular accumulation of endocytosed BSA-Alexa and RBP-Alexa within proximal tubule cells (Lee et al., 2013). To determine whether lysosomal degradative capacity was altered, Lee and colleagues also evaluated the expression of lysosomal proteases following albumin overload (Lee et al., 2013). They observed increased cathepsin L expression, whereas cathepsin B expression remained unchanged, suggesting a lysosomal adaptive response to albumin overload. Similarly, Olbricht and colleagues (1990), based on cathepsin activity assays in isolated proximal tubules from proteinuric rats, proposed that increased cathepsin B and L activities represent an adaptive response to increased protein uptake. Together, these findings suggest that intralysosomal degradative capacity remains largely preserved under protein-overload conditions (Olbricht et al., 1990). Therefore, intracellular albumin accumulation may instead result from impaired vesicular trafficking and defective delivery of endocytosed albumin to lysosomes. Based on this rationale, the reduction in BSA-FITC degradation observed in the present study is likely associated with defects in early post-endocytic events rather than impaired lysosomal degradation itself. Specifically, albumin overload may compromise the progression of early endosomes toward late endosomes and lysosomes, where degradation normally occurs. Despite the limitations of the present study, our findings suggest that albumin-induced downregulation of CFTR may contribute to impaired endosomal acidification. This defect could hinder endosomal maturation and trafficking toward lysosomes, thereby reducing albumin delivery to degradative compartments and promoting the accumulation of BSA-FITC within extra-lysosomal intracellular compartments. It is important to acknowledge that the BSA-FITC fluorescence decay assay, while widely used in proximal tubule cell studies to assess endolysosomal function (Gekle et al., 1995; Ishibashi, 2006), cannot unambiguously distinguish between defective endosomal acidification, impaired vesicular trafficking, delayed lysosomal delivery, and reduced proteolytic activity. Future experiments using direct pH measurements with ratiometric fluorescent probes or genetically encoded pH sensors, combined with co-localization analyses of internalized BSA-FITC with compartment-specific markers for endosomes and lysosomes, will be essential to identify the specific trafficking step affected by albumin overload and to determine the contribution of impaired endosomal acidification to the observed defects in albumin processing.

The BSA-FITC degradation experiment at 48 h was prioritized because this time point was associated with the most pronounced functional phenotype, including both CFTR and V-ATPase downregulation and higher reduction in BSA-FITC uptake, providing the best conditions to detect an impairment in albumin processing. The 24 h time point, at which only CFTR (but not V-ATPase) was reduced and the uptake inhibition was milder, was de-prioritized based on these considerations. Including BSA-FITC degradation data at 24 h, when CFTR is already reduced but V-ATPase expression is not yet significantly affected, would provide important information about whether CFTR downregulation alone is sufficient to impair albumin processing, and represents an important aim for future experiments.

This proposed mechanism is further supported by the BSA-FITC uptake experiments. The magnitude of uptake inhibition was considerably greater after 48 h of treatment, a time point at which both CFTR and V-ATPase expression were reduced. While exposure to high albumin concentrations for 24 h resulted in an approximately 14% reduction in BSA-FITC uptake, treatment for 48 h produced an approximately 44% reduction. These findings suggest that prolonged exposure to albumin overload promotes a synergistic effect of CFTR and V-ATPase downregulation, leading to a greater impairment of proximal tubule endocytosis. The BSA 0.1 group was not included in the BSA-FITC endocytosis analysis, as CFTR and V-ATPase expression levels under this condition were not significantly different from those of control cells (CTRL) at either time point. This absence of a protein expression phenotype at the low-dose albumin concentration suggests that endocytic alterations driven by the CFTR/V-ATPase axis would not be expected at 0.1 mg/mL. Nevertheless, future dose-response experiments encompassing a range of intermediate albumin concentrations should include this concentration to fully characterize the dose-dependent effects on endocytic function.

Future experiments involving CFTR overexpression under albumin-overload conditions or pharmacological CFTR activation will be essential to establish a direct causal relationship between CFTR downregulation and the observed defects in albumin endocytosis and degradation. The development and validation of a functional CFTR overexpression system in LLC-PK1 cells, however, presents significant technical challenges given the complexity of CFTR biogenesis and its requirement for proper co-translational folding and ER quality control (Pranke and Sermet-Gaudelus, 2014), and is planned as a specific aim of future investigations.

The present study demonstrates that exposure of porcine proximal tubule cell line LLC-PK1 to a high albumin concentration leads to a coordinated reduction in CFTR and V-ATPase expression, accompanied by impaired albumin endocytosis and intracellular degradation. These changes were associated with no major alterations in CFTR overall subcellular distribution. We propose that the downregulations of CFTR and V-ATPase are associated with impaired albumin endocytosis and intracellular processing and may represent a potential mechanism contributing to the worsening of albuminuria and the progression of kidney disease under proteinuric conditions (Figure 5).

**FIGURE 5:**
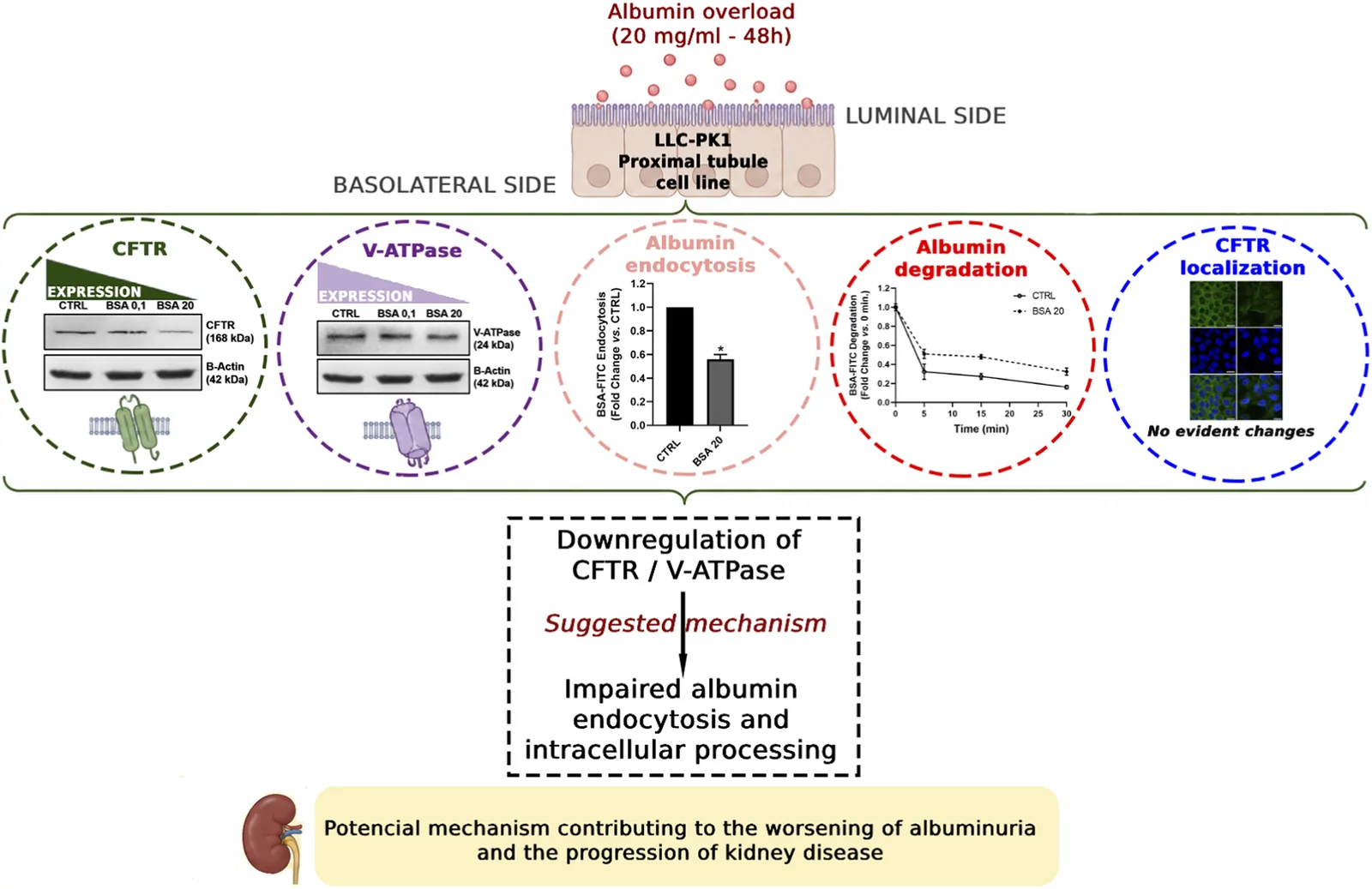
Albumin overload impairs albumin handling in proximal tubule cells through the downregulation of CFTR and V-ATPase. Western blot analyses demonstrated reduced expression of CFTR and V-ATPase compared with control cells. Functional assays revealed a significant decrease in albumin endocytosis and impaired intracellular albumin degradation. Despite the reduction in CFTR expression, confocal microscopy showed no evident changes in its subcellular localization. Based on these findings, we propose that the downregulation of CFTR and V-ATPase is associated with impaired albumin endocytosis and intracellular processing and may represent a potential mechanism contributing to the worsening of albuminuria and the progression of kidney disease under proteinuric conditions. The graphs show the experimental data obtained in the present study, whereas the schematic illustration summarizes the proposed mechanistic model.

## Data Availability

The raw data supporting the conclusions of this article will be made available by the authors, without undue reservation.
